# Scan-Rescan Reproducibility of High Resolution Magnetic Resonance Imaging of Atherosclerotic Plaque in the Middle Cerebral Artery

**DOI:** 10.1371/journal.pone.0134913

**Published:** 2015-08-06

**Authors:** Xuefeng Zhang, Chengcheng Zhu, Wenjia Peng, Bing Tian, Luguang Chen, Zhongzhao Teng, Jianping Lu, Umar Sadat, David Saloner, Qi Liu

**Affiliations:** 1 Department of Radiology, Changhai Hospital, Shanghai, China; 2 Department of Radiology and Biomedical Imaging, University of California San Francisco, San Francisco, CA, United States of America; 3 Department of Radiology, University of Cambridge, Cambridge, United Kingdom; 4 Cambridge Vascular Unit, Addenbrooke’s Hospital, Cambridge Biomedical Campus, Cambridge, United Kingdom; Medstar Washington Hospital Center, UNITED STATES

## Abstract

**Purpose:**

To evaluate the scan-rescan reproducibility of high-resolution magnetic resonance imaging (MRI) of middle cerebral artery (MCA) plaque, and calculate the number of subjects needed for future longitudinal clinical studies.

**Material and Methods:**

Twenty two patients with MCA plaque were scanned twice by a T_2_-weighted fast-spin-echo sequence at 3T. Areas and volumes of MCA lumen, total vessel and plaque were quantified and compared between two repeated scans. Agreement and measurement error was quantified by intraclass correlation coefficient (ICC) and coefficient of variance (CV) as defined by standard deviation (SD) of pair wise difference / mean. Sample size needed to detect 5% to 20% changes in area/volume was calculated using 80% power and 5% significance level.

**Results:**

There was no significant different between the area and volume measurements of two repeated scans (p>0.05) with good agreement (ICC range 0.97–0.98 for area and 0.99 for volume). Relatively small measurement errors were observed with CVs range 6.1%-11.8% for area quantification and 4.9%-8.0% for volume quantification. Volume measurements tended to have 19.7% to 32.2% smaller CVs compared with area measurements. Sample size calculation showed a group of 47 patients was sufficient to detect 5% to 10% changes in MCA area/volume.

**Conclusion:**

High resolution MRI is feasible for quantifying intracranial plaque area and volume in longitudinal clinical studies with low scan-rescan variability. Volume measurement tends to be more reproducible compared with area measurements.

## Introduction

Intracranial large-artery atherosclerotic disease has a large worldwide burden with a high prevalence in Asian, Hispanic and African races (6–56%) [1]. Among them, middle cerebral artery (MCA) is one of the most prevalent territories that are affected, and patients with symptomatic MCA stenosis have an overall stroke risk of 12.5% per year [2].

Assessment of intracranial arterial stenosis based on digital subtraction angiography is well established. However, luminal evaluation alone is insufficient to assess the disease severity as it fails to provide information on the underlying pathology within the wall [3]. Two (2D) and three dimensional (3D) high resolution black blood magnetic resonance imaging (hrMRI) has shown to be a promising tool for imaging intracranial vessel wall due to its non-invasiveness and excellent soft tissue contrast [3–9]. Small size retrospective studies have shown that high-risk plaque features, such as large plaque burden, intra-plaque hemorrhage and post-contrast enhancement, could differentiate symptomatic and asymptomatic groups [4–9]. Despite the small size and deep location posing a great challenge in the intracranial plaque imaging, the development of hrMRI techniques provides opportunities in longitudinal clinical trials to understand the disease progression and optimize the treatment strategy.

With this relevance, the reliability of hrMRI in characterizing the intracranial atherosclerosis needs to be assessed. Recently Yang et al. found good intra- and inter-observer agreement in quantifying MCA wall areas and identifying plaque components and enhancement using multi-contrast hrMRI [10]. However, the scan-rescan reproducibility of this imaging technique remains predominantly unexplored. This study, therefore, aims to: (a) study the scan-rescan reproducibility of hrMRI in quantifying areas and volumes of atherosclerosis in MCA; and (b) calculate the sample size needed for future longitudinal clinical studies.

## Methods

### Study population

The study was conducted following approval of Shanghai Changhai Hospital Ethics Committee (CHEC2013-204). Written informed consent was obtained from all patients. Twenty-two patients (18 male, age 55±11, 3 diabetes, 7 hypertension, 9 hyperlipidemia and 8 smokers) were recruited in this study with inclusion criteria of: (1) presenting neurological symptoms with MCA territory infarcts identified clinically or by MRI/CT brain imaging; (2) absence of significant carotid arterial stenosis (<30%) assessed by ultrasound; (3) absence of atrial fibrillation on 24hr monitoring; (4) absence of ascending aortic arch atheroma on MR; (5) absence of non-atherosclerotic intracranial arterial disease including inflammatory arteritis and congenital agenesis; and (6) absence of total MCA occlusion. Patients with pacemakers, certain types of metallic implants, severe claustrophobia were excluded.

### MRI acquisition

**Fig 1:**
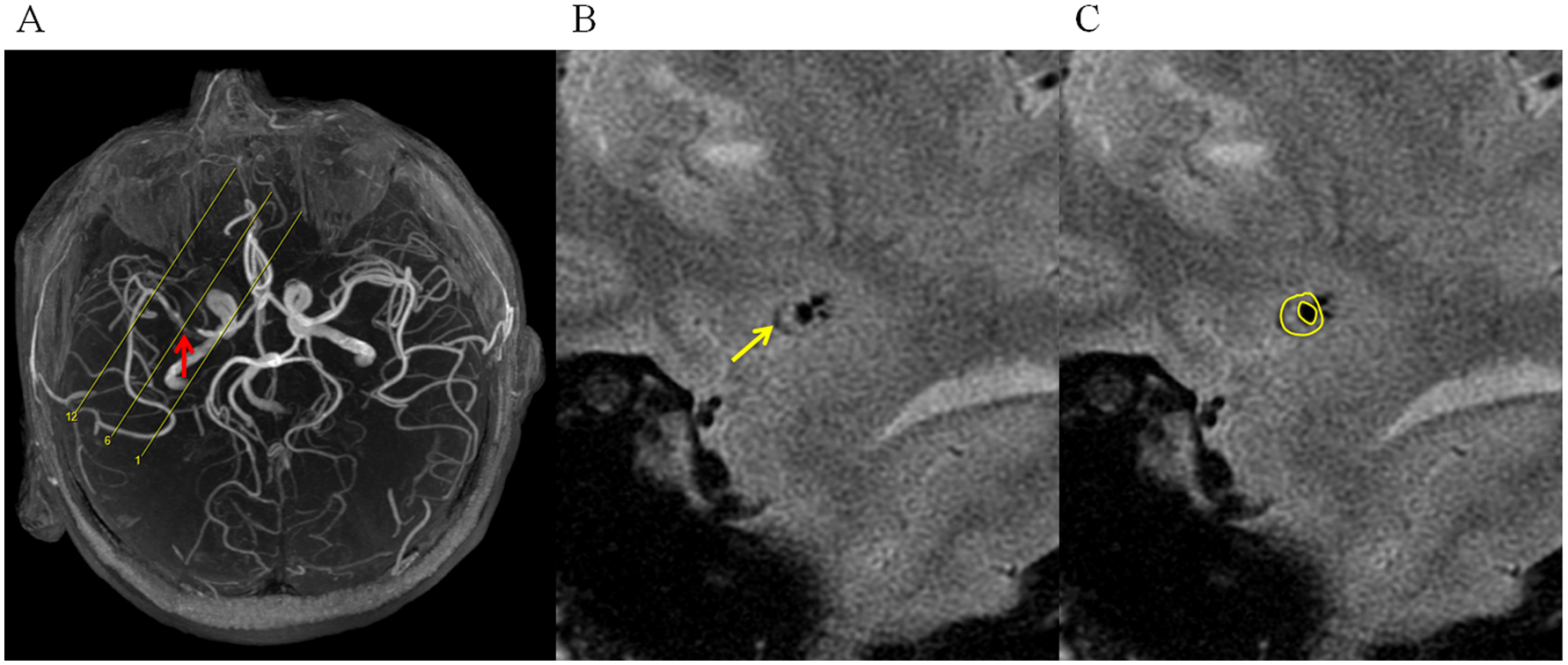
Image acquisition and segmentation of middle cerebral artery (MCA) plaque. (A) Prescription of slices perpendicular to MCA base on the maximal intensity projection (MIP) of 3D TOF images. Red arrow shows the stenosis site. (B) Sample images (slice number 6) shows MCA plaque (yellow arrow). (C) Segmentation of the MCA lumen and outer wall boundaries.

Imaging was performed on a 3T whole body MRI system (GE Signa 3.0T HDxt, GE Healthcare, Waukesha, WI, USA) using an 8-channel phased-array head coil by a MR scientist (LC). Patients were scanned twice by a two dimensional (2D) high-resolution black blood T_2_-weighted fast-spin-echo (FSE) sequence. After an initial multi-plane localizer sequence, axial 3D time-of-flight (TOF) MR angiography was performed to identify the location of the MCA stenosis. Then the T_2_-weighted FSE sequence was prescribed with slices perpendicular to the MCA based on the maximal intensity projection (MIP) of TOF images (Fig 1a). A saturation band was prescribed parallel to the slice direction and proximal to the inlet of MCA in order to suppress the blood signal. Scan parameters: TR/TE = 2884ms/51ms; 12 slices with 2mm slice thickness and 0.5mm gap; flied of view (FOV) 10cm×10cm; no phase wrap; matrix 256×320; in-plane resolution 0.39mm×0.31mm; echo train length (ETL) 20; scan time 3 minutes and 45 seconds; number of averages 6. After the initial scan, patients were asked to get off the scanner table, have a rest for 5–10 minutes, and then get back on the table again for the second scan with the same imaging protocol.

### Image analysis

One patient was excluded from the analysis due to significant motion artifacts. In total, 69 pairs of slices (scan-rescan) covering atherosclerotic lesions from 21 patients were analyzed. The analysis was performed by a single reviewer (XZ). The reviewer firstly analyzed the first scan images of all patients. Then the rescan images were sorted in a random order and analyzed by the same reviewer. Lumen and outer wall boundaries were manually segmented using CMR tools (Cardiovascular Imaging Solutions Ltd, London, UK) and their areas were calculated. Plaque area (PA) was defined as the difference between total vessel area and lumen area. Volumes of lumen (LV), vessel (VV) and plaque (PV) were characterized as the product of areas, number of slices and slice thickness.

### Statistical analysis

Normality assumptions were assessed using a Shapiro-Wilk’s test. Distributions were summarized using the median [interquartile range (IQR)]. A paired t-test or Wilcoxon matched paired test was used where appropriate. Differences between areas/volumes of two repeated scans were assessed using the Bland–Altman analysis [11]. The mean of the pair wise differences was reported as bias and the 95% limits of agreement [LOA; LOA = bias±1.96×SD (standard deviation)]. The intra-class correlation coefficient (ICC) was used to measure the agreement between two scans. Measurement error between scans was quantified by coefficient of variance (CV; CV = SD/mean×100%). For each measurement, sample size calculation was based on a two sample unpaired t-test with 80% power and 5% significance level (two sided) as adopted from a previous publication [12]. Sample size needed to detect 5%, 10%, 15% and 20% changes in each measurement was calculated. Statistical analyses were performed in R 2.5.1 (The R Foundation of Statistical Computing, Vienna, Austria).

## Results

**Fig 2:**
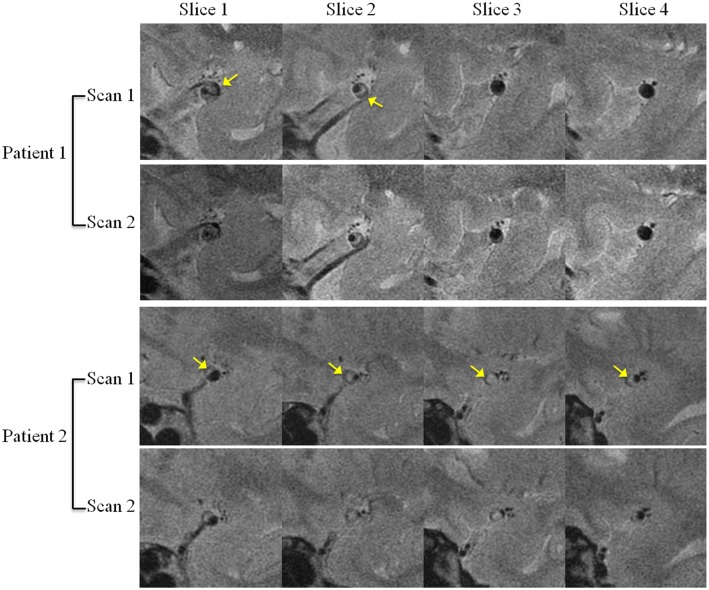
Sample scan and re-scan images of middle cerebral artery plaques from two patients. Arrows show the plaques.

**Fig 3:**
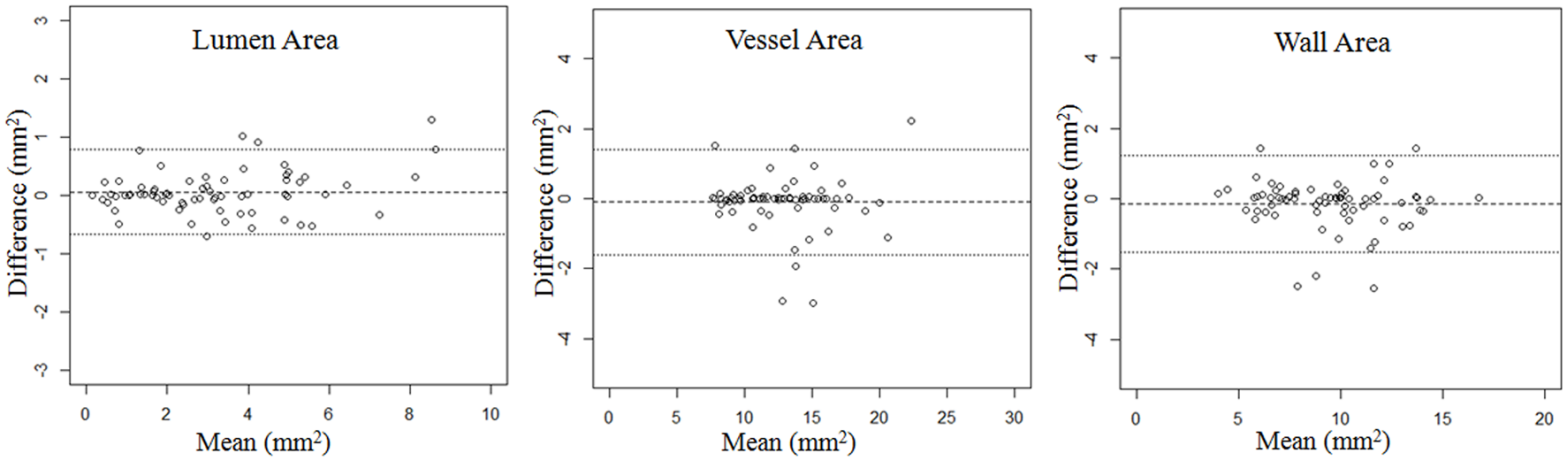
Bland-Altman plot of area measurements between two repeated scans. Solid lines define bias, dashed lines define limits of agreement (LOA).

**Fig 4:**
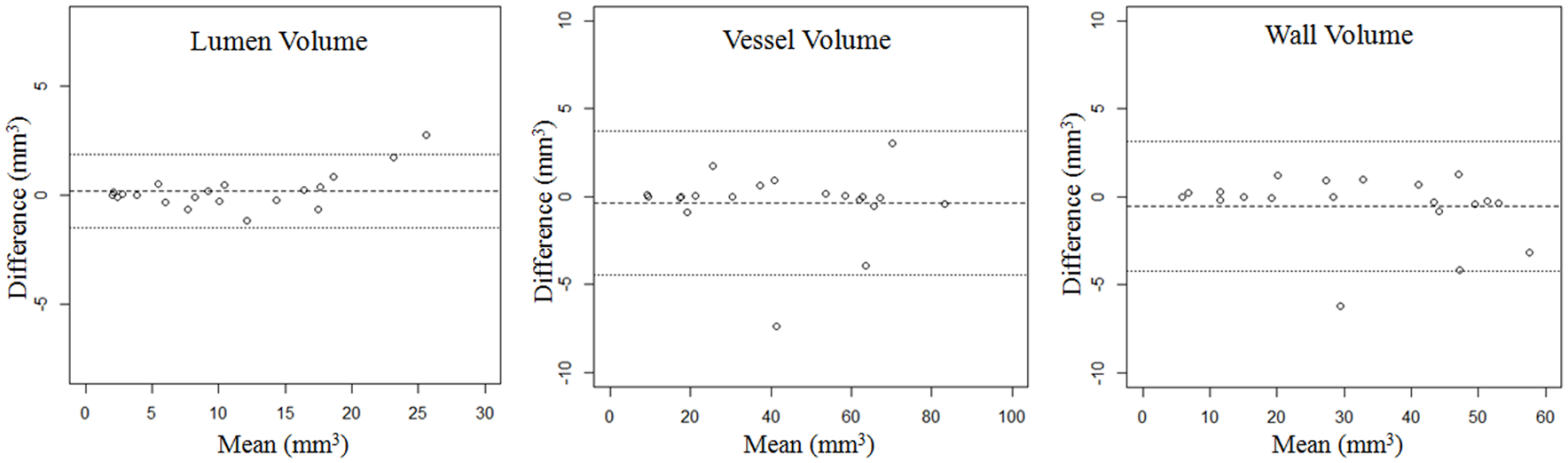
Bland-Altman plot of volume measurements between two repeated scans. Solid lines define bias, dashed lines define limits of agreement (LOA).

As shown in Table 1, there was no significant different between the area and volume measurements of two repeated scans (p>0.05). Representative matched images from two patients were shown in Fig 2. Excellent agreement were found in various measurements from two scans with ICC>0.97 (Table 2). Bland-Altman analysis for area and volume of lumen, vessel and plaque of two repeated scans were shown in Figs 3 and 4. The bias, LOA and ICC values were summarized in Table 2. No significant bias was noted between two scans, and there was no trend observed between magnitudes of the measurements with the variance.

**Table 1:** Comparison of area and volume measurement between two repeated scans. Data are summarized as median [inter quartile range].

	Scan 1	Scan 2	p
Lumen Area (mm^2^)	3.00 [1.71, 4.69]	2.87 [1.63, 4.44]	0.49
Plaque Area (mm^2^)	9.42 [6.98, 11.08]	9.66 [6.99, 11.65]	0.08
Vessel Area (mm^2^)	12.40 [10.09, 14.42]	12.64 [10.00, 14.71]	0.67
Lumen Volume (mm^3^)	9.58 [5.22, 16.66]	9.64 [4.84, 16.56]	0.60
Plaque volume (mm^3^)	30.08 [18.09, 45.80]	32.38 [18.17, 47.16]	0.65
Vessel Volume (mm^3^)	39.62 [20.67, 62.26]	42.85 [20.86, 63.47]	0.89

**Table 2:** Reproducibility analysis between two scans.

	Mean	SD(between scan)	CV	bias	LOA	ICC
Lumen Area (mm^2^)	3.14	0.37	11.8%	0.05	(-0.67, 0.78)	0.98
Plaque Area (mm^2^)	9.37	0.70	7.5%	-0.15	(-1.52, 1.22)	0.97
Vessel Area (mm^2^)	12.56	0.77	6.1%	-0.10	(-1.60, 1.41)	0.97
Lumen Volume (mm^3^)	10.68	0.85	8.0%	0.18	(-1.50, 1.85)	0.99
Plaque volume (mm^3^)	31.84	1.88	5.9%	-0.52	(-4.21, 3.17)	0.99
Vessel Volume (mm^3^)	42.70	2.08	4.9%	-0.34	(-4.42, 3.74)	0.99

SD: standard deviation; CV: coefficient of variance; LOA: limit of agreement; ICC: intra-class coefficient.

Means and SDs of area and volume from the two scans were summarized in Table 2. Small CVs were found for both area (6.1%-11.8%) and volume measurements (4.9%-8.0%). However, volume measurements tend to have smaller variances compared with area measurements (CVs were 19.7% to 32.2% smaller). Lumen area and volume measurements had the largest error, while the total vessel area and volume measurements had the smallest error.

Sample size calculation for detecting 5% to 20% change in area and volume was summarized in Table 3. A sample size of 47 allowed the detection of 5% change in vessel area and volumes of vessel and plaque, and 10% change in both area and volume measurements. A smaller sample size of 18 could detect 10% changes in plaque area and volume. In general, the use of volume measurements as an end point required smaller sample sizes compared with area measurements.

**Table 3:** Sample size calculation for detecting 5% to 20% change in area and volume. (Power: 80%, p = 0.05).

	CV	5%	10%	15%	20%
Lumen Area	11.8%	176	44	20	11
Vessel Area	6.1%	47	12	6	3
Plaque Area	7.5%	72	18	8	5
Lumen Volume	8.0%	81	21	9	6
Vessel Volume	4.9%	31	8	4	2
Plaque volume	5.9%	44	11	5	3

CV: coefficient of variance.

## Discussion

In this study, we demonstrated a good scan re-scan reproducibility of hrMRI in quantifying area and volume of atherosclerotic plaque in MCA, and provided the sample sizes estimation for planning future longitudinal clinical studies. A sample size <50 was found to be sufficient to detect 5% to 10% changes in area and volume. Volume measurements tended to have smaller measurement error compared with area measurements, which requires less sample size in longitudinal studies. To our knowledge, this is the first study to assess the scan re-scan reproducibility of hrMRI for intracranial atherosclerosis imaging and provide sample size calculations for longitudinal studies.

Recently Yang et al. studied the reader agreement of plaque area measurements and component/contrast enhancement characterization in MCA using multi-contrast hrMRI at 3T [10]. Good intra-observer (ICC range 0.95–0.97) and inter-observer agreement (ICC range 0.87–0.96) was found for area quantifications, suggesting that hrMRI could be a reliable tool for MCA plaque imaging. Except for reader variability, the scan re-scan reproducibility is an important parameter assessing the reliability of such imaging technique as errors may be introduced during re-scan, including instrumental factors, patient/coil positioning and slice orientations/co-registration. Experiences from carotid plaque imaging suggested that the major factor affected reproducibility was slice orientations and image co-registration [13], mostly due to the anisotropic voxels. In this study, during each scan, the slices were prescribed carefully in coronal and axial MIPs of TOF images to make sure they were perpendicular to the vessel to minimize the effect of slice orientation. In addition, 3D TOF images were used to assist the co-registration. Compared with volume, the larger error in area measuring observed in this study may also attribute to the slice orientation and location mismatch despite the effort to minimize the effect from these two aspects. For the volume measurement, such error was reduced as multi slices were considered and the perturbations might be averaged. Considering this relevance, volume maybe more sensitive compared with area in longitudinal clinical studies to detect plaque changes. Moreover, as shown in Table 3, a smaller sample size is needed when volume is used. Furthermore, compared with area, volume may be a better parameter to characterize plaque burden as it grows longitudinally.

In this study, the largest error was found in lumen area measurement and smallest for outer wall. That might be due to the small size of luminal area. As a result, a small absolute difference in lumen segmentation would lead to a big relative error. With the same reason, as intracranial artery is much smaller than carotid artery, the CVs obtained in this study were larger than those based on carotid imaging (7.1%-9.8% for area measurement and 2.3%-5.8% for volume measurement [12] [14]). Imaging using machines with a higher magnetic strength, such as ultra-high 7T MRI, may be able to achieve a better reproducibility [9]. In addition, flow artifact induced by the tortuous configuration of MCA may attribute to the error in lumen segmentation therefore leading to a big CV. The use of more advanced blood suppression techniques [15] [16] may overcome this limit and improve the accuracy and reproducibility in the lumen delineation. The reproducibility could be further improved when 3D isotropic MR sequences are used. Recently developed variable flip angle FSE sequences have enabled high isotropic resolution (~0.5mm isotropic) in intracranial plaque imaging [8] [17]. The mismatch in slice location and orientation could be minimized using 3D volumetric information reconstructed based on isotropic voxels. However, such techniques need long scan time and may induce edge-enhancement artefacts and blurring due to the long echo train [18] [19]. Therefore, future optimization and validations of such techniques are needed.

One limitation of this study is the short scan gap between two scans (5–10 minutes). Even though the patients were repositioned and the slices were prescribed again on a different localizer at the second scan, the operator may partially remember the previous setup. Therefore, such protocols may underestimate the variance between scans in a true clinical setting. However, as the slices were prescribed based on axial and coronal MIPs of 3D TOF images and the slice orientation error was already minimized, we believe such limitation did not influence the major conclusions of this study.

## Conclusion

High resolution MRI is feasible for quantifying intracranial plaque area and volume in longitudinal clinical studies with a small scan re-scan variability. A sample size of 47 is able to detect 5% to 10% changes in MCA area/volume. Volume measurements tend to be more reproducible compared with area measurements.
